# Transforming Primary Care: Developing Health Precincts as Models for Sustainable Integrated Community-Based Healthcare

**DOI:** 10.3390/healthcare11050673

**Published:** 2023-02-24

**Authors:** Florin Oprescu, Shauna Fjaagesund, Margaret Hardy, Evan Jones

**Affiliations:** School of Health, University of the Sunshine Coast, Sippy Downs 4556, Australia

**Keywords:** health precinct, health hub, primary healthcare, community-based healthcare

## Abstract

Holistic healthcare precincts are an emerging service model to address the growing health service demands of ageing consumers and an increasing prevalence of chronic diseases. In Australia and similar countries with universal publicly funded Medicare systems, the first point of access to healthcare is provided by general medical practitioners. This case report focuses on successful components of a private, integrated, patient-centred primary care model located in a low socioeconomic population in North Brisbane, Queensland. Successful components included a focus on sustainability, general practice as an anchor tenant in the health precinct, the integration of multiple services, team-based care for shared clinical services, flexible expansion options, the use of MedTech, support for small businesses and a cluster structure. The Morayfield Health Precinct (MHP) offers appropriate, safe and individualised healthcare to residents across their life continuum. Its success was built on a foundation of pre-planning, to ensure the design/build, anchor tenant and collaborative ecosystem were sustainable in the long term. MHP planning was based on an adaptation of the WHO-IPCC framework supporting true patient-centred, integrated care. Its shared vision and collaborative care are supported by its internal governance structure, tenant selection, established and emerging referral networks and partnerships. Evidence-based and informed care is further supported by internal and external research and education partnerships.

## 1. Introduction

Holistic healthcare precincts are an emerging service model to address the growing health service demand of ageing consumers and an increasing prevalence of chronic diseases. Innovative patient-centred approaches and integrative place-based healthcare infrastructure are being developed to create environments that support better individual and community health outcomes [1,2]. Integrated services should be tailored to the needs of each health consumer and across different life stages [3], including rapid and efficient referral pathways for those with complex needs requiring team-based care [4]. In Australia and similar countries with universal publicly funded Medicare systems, the first point of access to healthcare is provided by general medical practitioners [5]. Globally, one in five adults who required the services of a general medical practitioner (GP) did not receive care, with priority groups such as people of low socioeconomic status experiencing higher levels of inaccessibility [5]. The World Health Organization (WHO] encourages all countries to support universal affordable health coverage and access to a range of essential services to reduce poverty, increase economic opportunity and create more stable communities [6]. Integrated healthcare systems can support quality affordable comprehensive care for everyone, including broader social determinants of health and the empowerment of individuals and communities [3,6]. During the COVID-19 pandemic, policy measures were introduced to provide Australians with access to primary care. General medical practitioners were described by patients as most accessible, either face-to-face or by telehealth. Often, however, other multidisciplinary team members were not available, such as non-urgent specialists, allied health, dental care services and in situations in which telephone appointments were viewed as inappropriate by a patient or practitioner [7]. Sustainable health systems are necessary during pandemics to support individualised patient decisions in holistic health, safety and complex care, including initiatives that provide reassurance of infection safety and continued engagement with all multi-disciplinary team providers [8].

This case report focuses on successful components of a private, integrated, patient-centred primary care model located in a low socioeconomic population in North Brisbane, Queensland, including its ability to provide multidisciplinary care during the COVID-19 pandemic. Though the initial facility opened in 2017 and was labelled a ‘Health Hub’, it was developed and built to the capacity of a large primary care health precinct [9]. Its success can be measured by its number of growing health consumers, which has increased to more than 200,000 current patients on record [10]. This is quite significant, given the immediate suburb of Morayfield has ~60,000 residents and is centrally located within the local government area of Moreton Bay with a population of 476,340 [11,12]. In 2021, MHP was sold by its original private investors to a large real estate corporation for AUD 110 M [13].

Thus, the proposed research question (RQ) is: What are the elements of the successful planning and implementation of a sustainable and adaptable large community-based integrated healthcare hub? The results of this case study are intended to broadly present a novel model of integrated care in primary care and elements of successful planning and implementation to inform area(s) of future evaluation.

## 2. Methods

A case study approach was used to answer the RQ, as limited literature exists in Australia on emergent, privately funded, community-based, primary care health precincts. The selected case was chosen due to its unique elements of: (1) its rapid expansion and large health consumer base; (2) its infrastructure and governance investment, which was privately funded with no government support; (3) its focus on primary multi-disciplinary care opposed to a hospital-based setting; (4) its location in a low-socioeconomic area with a low uptake of private health insurance; and (5) its sustainable and adaptative model based on engagement. Supplementary information, such as governance committee minutes, was provided. Limitations to this case study include the fact that the results were based on the retrospective information made available to authors.

## 3. Results

### 3.1. Planning Development to Meet Community Needs

The MHP was purpose-built and designed to serve a population of low socioeconomic status in Moreton Bay North that was reported to have one of the poorest health outcomes [14,15]. Firstly, during the initial planning stage, a comprehensive community needs analysis was undertaken to inform which health services, including treatment modalities, met the expressed and unexpressed health needs of residents. This analysis took into consideration local population demographics and health status, which included a high prevalence of chronic disease, multimorbidity, disability, homelessness and households living in the lowest two quintiles of disadvantage (including poverty and crime). Secondly, asset mapping was conducted to inform which identified health needs were undelivered to low socioeconomic consumers within their community. Prior to the opening of the MHP, residents had to travel long distances for specialist services, which reduced accessibility and affordability [16].

### 3.2. Implementation of Evidence-Based Community Services

Built for sustainability. Health consumer and asset pre-planning data informed an initial facility of 15,000 sqm to accommodate the diversity and clustering of health services needed (Table 1). Other important considerations included proximity to public transport, accessibility by car and adequate free parking and loading areas to reduce travel barriers. An existing large retail structure (the former Bunnings building) was purchased by investors and retrofitted to reduce environmental impact to the community. To further MHP’s commitment to healthier communities, investment in 404 kW solar generation, rainwater recycling, electric vehicle charging, and facilities to encourage active transport were also included in the building design.

Primary care access. The anchor tenants within the MHP are general medical practitioners (GPs) who provide multi-dimensional care to residents. This care delivery encompasses the emotional, physical, cultural, environmental, spiritual and other non-biomedical factors unique to the individual person, built on the foundation of a doctor–patient relationship that can provision team-based care [17]. To create added convenience and reduce the burden of multiple appointments, a range of practitioners and complementary services was positioned within and adjacent to MHP to support an integrated, patient-centred, team-based care environment (Table 1). In alignment with precinct pre-planning, services were expanded to include a range of specialists to support diagnosis and treatment (Table 1).

COVID-19 response. During the COVID-19 pandemic, Australian patients with respiratory illness were faced with disruptions in primary care and access to their regular general medical practitioner (GP) due to concerns about infection safety [18]. The MHP patients experienced limited disruptions due to its collective tenant initiatives, which increased assurance of the safety of routine care appointments. This included a tenant investment in the front entrance screening, cleaning schedules, the alignment of workplace policies and procedures, provision of masks and external marketing to reassure that safe and appropriate care was accessible. Additionally, MHP was the first COVID-19 respiratory clinic to open in Queensland due to this preparedness, as well as its existing physical design of a quarantine clinic area with its own separate air exchange and access. This fit-for-purpose approach continued in 2021, with the custom design of a mass vaccination clinic that had a capacity of 7000 patients per week.

Sustainability of integration. The MHP uses an adaptation of the WHO Integrated Person-Centred Care (WHO-IPCC) framework [3], which includes additional sustainability measures [19] to maintain a shared vision of delivering true patient-centred care as a collective precinct of autonomous tenants. Shared values are supported by an overarching strategic plan, governance structure and tenant investment, which was initially developed by all stakeholders, including health consumers. Elements of the Canterbury Model of Care [20] and Kaiser Permanente [21] were integrated into MHP objectives to deliver coordinated, appropriate, safe and tailored individual care to health consumers. Continuing tenant investment in MHP’s governance is a point of differentiation from other precincts, such as the General Practice Super Clinics [22]. Health consumers are encouraged to be active participants. Examples include research co-design, quality improvement feedback and connection to community services.

Team-based care. Multidisciplinary care delivery is underpinned by governance, tenant selection and rental agreements, which ensures managers, practitioners and support staff work together to deliver coordinated, individualised care. This coordination of clinical and non-biomedical services, including community-based services that connect patients to employment, training and social activities are supported by trusted referral networks based on established relationships created by interaction, proximity and health consumer feedback.

Tenants with regular, established referrals with other providers within MHP have some level of shared patient records or processes in place, ensuring that consent is received and privacy is protected. Regular clinical meetings are held quarterly, bi-monthly and weekly [depending on each committee’s terms of reference], bringing all MHP tenants and practitioners together to ensure open communication, build referral networks and improve patient experiences. Evidence-based and informed care is based on the pillars of education, research and training as foundations of good clinical practice, which supports the rapid translation of research into improve patient health outcomes.

Novel delivery of shared clinical services. Some tenants within MHP choose to share space and resources (a practice within a practice) to deliver seamless care to health consumers. For example, the community pharmacy has multiple treatment rooms to deliver additional services, such as immunisations. This includes all COVID-19 vaccine types, as well as both private and public influenza [23,24]. In Australia, this approach is particularly novel, as throughout the pandemic, pharmacies and/or general practices were at times restricted to the administration of certain types of COVID-19 vaccines. This shared delivery of services better accommodates health consumer preferences, as it brings vaccine services together, within the same location. Additional benefits for health consumers include better access to multiple health practitioners, increased safety in the event of anaphylaxis and the opportunity for multi-disciplinary holistic health guidance at routine vaccination appointments.

Novel team-based chiropractic care. Another novel integration includes an embedded chiropractic care service within primary care. This service is delivered in partnership between multiple MHP tenants using a shared patient record within a trusted referral network to provide evidence-based team care for health consumers experiencing chronic pain [25]. Allied health services within and adjacent to MHP provide additional care plan support, in addition to group and peer-based exercise activities to reduce the cost burden to health consumers. The regional council also provides free exercise and social activities to residents [26], which practitioners refer and partner with to greater assist patients in selecting activities based on their preferences.

Urgent care expansion. A current expansion focused on immediate care services is in its pilot phase [27]. The Minor Accident and Illness Centre (MAIC) and medical practitioner workforce was accredited to the New Zealand College of Urgent Care (NZCUC, 2021), as no Australian equivalent exists. This 38-bed centre provides acute services for non-life-threatening accidents and illnesses and its co-location within MHP supports continuity of care (i.e., follow-up with a family doctor, specialist, counsellor or psychologist).

Due to the low-resource, high complex care population demographics of North Moreton Bay [11,12], it is important that acute services are provided at no cost to patients. This differs from private emergency and urgent care services found in more affluent suburbs of Australia. Onsite pathology and radiology are also both integral elements of a successful urgent care model, as they provides health consumers with immediate diagnosis and treatment. In other countries, urgent care in certain settings can reduce hospital wait times and congestion for minor illnesses and accidents when appropriately and safely treated in an accredited and qualified urgent care centre [28,29].

Assistive technology. Emerging MedTech compliments and gives resources to practitioners in the prevention, early diagnosis and/or treatment of illness and chronic disease. Within MHP, this includes artificial intelligence and/or machine learning for ap-plications such as mole mapping to detect skin cancer or lesion changes [30], sound detection for diagnosis of respiratory illnesses [31] and tailored repetitive magnetic stimulation to improve health outcomes of people living with neurological disorders [32]. Novel chronic disease management mobile apps connect patients to services to improve access to health and wellbeing activities [24].

The MHP pharmacy has a robotic dispensary, which greatly improves operational efficiencies by removing burdensome physical dispensing and administrative tasks [24,33]. This permits pharmacists to be a single point of transaction and significantly increases the counselling time given to health consumers [33,34]. Additional pharmacist time with patients builds trusted relationships, assists with the identification of risks and safety compliance in treatment and helps develop interprofessional relationships with practitioners.

Health incubator. Smaller tenants may start within MPH and grow to relocate adjacent to the precinct or within the local community. These tenants remain part of the MHP referral networks and are invited to remain active participants in MPH activities. Examples include atWork Australia, which connects residents to training and meaningful employment [35], and Peach Tree, a peer-led organisation that supports perinatal resilience and recovery [36].

Cluster structure. From a cluster ecosystem model, the MHP consists of a mental health cluster, including community addiction and relationship services; a muscoskeletal cluster supported by complimentary services; a skin cancer cluster with increasing assistive MedTech support; Paeds (Mums and Bubs Hub), including a focus on interventions in pre-birth and early childhood; and chronic disease management due to the high needs and low socioeconomic status of the local population. More details are available in Table 1.

## 4. Discussion

The MHP offers appropriate, safe and individualised healthcare to residents across their life continuum, with clusters of services (Table 1) and referral pathways based on health consumer demand. Its success was built on the foundation of pre-planning, to ensure the design/build, anchor tenant and collaborative ecosystem were sustainable in the long term. MHP implementation was based on an adaptation of the WHO-IPCC framework, which continues to support an inclusive governance structure, based on the principles of sustainability, integrated care and patient-centredness.

It is broadly recognised that clinical leadership is an integral part of integrated care delivery [37]. Collective leadership, described as collaborative, shared, distributed or team-based, involves the engagement and influence of practitioners based on social interactions [38]. The MHP is supported by each tenant’s commitment and participation in internal governance as part of its adapted WHO-IPCC framework. Active participants within MHP’s ecosystem also include individual health practitioners, such as specialists, GPs, pharmacists, allied health practitioners, psychologists, counsellors and nurses. Evidence-based and informed care is further supported by internal and external research and education partnerships.

The proximity of services within the same location provides added convenience for health consumers, especially those with complex needs requiring multiple appointments and team-based care. This is demonstrated by the development of health clusters, including a significant presence of mental health support and muscoskeletal care. The creation of non-traditional integrative approaches, such as evidence-based chiropractors and the use of assistive medical technologies, further enhances holistic care by supporting early diagnosis, individualised treatment and self-management.

The physical design of the building facilitates activities and interactions between tenants and practitioners with a centralised education and training room. This space hosts weekly stakeholder-inclusive clinical meetings, which create established referral networks among tenants and individual practitioners. This continuing ecosystem of collaboration is based on engagement and a shared common vision, which also provides health consumers with reassurance that referred provider services are known to their practitioner and can be trusted.

The co-creation of community-based health systems involves academics working together with stakeholders to deliver societal impact, adaptative research processes and novel models (Table 2) to meet local needs [39]. In alignment with its adapted WHO-IPCC strategy, the Research Education and Engagement Committee [REEC] develops internal research capacity, builds bodies of evidence and supports the co-design and translation of research into clinical practice. This includes an expansion of health consumer representation as part of a newly formed Health Research Ethics Advisory Committee. Recently, a tenant investment of AUD 90,000 into seed research grant funding has facilitated initial relationships between industry, clinicians and researchers to collaborate on small projects. These studies are ongoing and are expected to leverage outcomes for future large-scale research projects [40]. This funding was especially important, as the MHP is not a hospital, and therefore, it receives no direct financial support from tertiary entities and is reliant on seed funding from tenancies and external research grants.

Sustainability was foundational for the MHP design and a key strategic pillar of MHP’s strategic plan. Financial sustainability, including the capacity to meet demand, was added to the WHO-IPCC framework to ensure scalability and flexibility to adapt to the needs of health consumers. This is an innovation, as sustainable services need to be financially sound in addition to being integrated to provide the best possible health outcomes for residents. To ensure the success of tenants, a continual review of community needs, workforce challenges and health consumer demand is undertaken at governance committees to drive new services and future tenant selection processes.

During the COVID-19 pandemic, the MHP was able to respond quickly to provide safe and accessible services to health consumers. By design, an area of the building was built with its own separate air exchange system and access. This permitted the MHP to establish the first COVID-19 respiratory clinic in Queensland, and from March 2020 to February 2023, it has provided over 157,000 appointments. By tenant agreement, additional risk mitigation and infection control strategies were collectively introduced, which included an investment front entrance screening and mask distribution to patients. These actions were taken to reduce COVID-19 exposure and increase patient confidence to attend routine care appointments.

Addressing current workforce shortages by the sharing of practitioners is enabled by co-location and engagement within the MHP ecosystem, as it permits separate tenants to provide services in partnership. Shared services bring mutual benefit, as this reduces unnecessary competition and duplication of services within the precinct, while also using existing space and workforce more efficiently. Additionally, working collaboratively in close proximity (i.e., nurses, doctors, pharmacists, technicians and medical administration) further develops interprofessional relationships and increases patient trust.

The expansion of practitioner roles could be integrated into emerging models of care to address primary care workforce shortages. This could include scope enhancement for nurse practitioners and pharmacists in non-hospital settings to support acute services and chronic disease management. Additionally, urgent care specialist training could be tailored across a range of health practitioner types (i.e., paramedicine, practice nurses, occupational therapists). These roles within team-based care and integration between providers requires further evaluation, in order to form recommendations on how to deliver quality care, reduce fragmentation and improve continuity of care within local communities.

## 5. Conclusions

MHP serves over 200,000 patients in an efficient, safe and appropriate manner (7 days/week, 12 h/day, 365 days per year). Its foundations are based on engagement in the community, business and clinical environment, and MHP’s shared common vision is embedded within planning and implementation processes. Both health consumers and practitioners experience the convenience and benefits of a physical co-location and the integration of needed health services. The collaborative ecosystem, supported by governance structures, is instrumental in creating innovation and addressing community health challenges, and further evaluation is needed to measure societal impact and patient health outcomes.

Tenant selection and committee processes are aligned with the shared vision of integration and patient-centeredness, including the foundational pillars of education, research and engagement. These pillars continue to support the delivery of evidence-based and -informed approaches, including novel models of care, to provide the best integrated (team-based) and patient-centred (individualised) care to health consumers. Further research into the interaction between clinical leadership in integrative care delivery and its emerging relationships with academics will be carried out.

Collaboration has supported tenants during periods of significant and unprecedented change. Tenants have demonstrated a surge capacity for pandemic situations. State-of-the art physical design and assistive technologies were further strengthened by collaborative processes and governance. The capacity for future developments that support the need for flexible and multi-sector coordination to address the changing needs of health consumers requires further evaluation to better determine how the components of this model improve patient health outcomes.

## Figures and Tables

**Table 1:** Morayfield health precinct cluster structure.

Cluster	Integrated Services
Mental Health	Counselling and psychology support Neuromodulation (tailored TMS) Peer group-based support or exercise Addiction and relationship support Holistic care and referrals to mental health services—GPs and nurse practitioners Allied health—dietetics and nutrition, exercise physiology, group/peer exercise support Onsite diagnostics Community pharmacy Access to evidence-informed treatment—clinical trials
Muscoskeletal	Chronic pain management—GPs and nurse practitioners Sports medicine specialists Complementary medicine specialists—prolotherapy, PRP, cannabinoids, peer group-based support/exercise Integrated chiropractic care Allied health—exercise physiology, physiotherapy, dietetics and nutrition, podiatry Rehabilitation specialists Community pharmacy Onsite diagnostics
Skin Cancer	Early diagnosis and prevention—GPs and nurse practitioners Oncology specialists GPs with specialist training in skin cancer Early detection tools—assistive medical technology in mole mapping, detection and changes Community pharmacy Onsite diagnostics Access to evidence-informed treatment—clinical trials
Paeds—Mum and Bubs Hub	Ante and post-natal holistic care—midwives, paediatricians and gynaecologists Lactation/breastfeeding support Sexual healthcare and referrals—GPs and nurse practitioners Neuromodulation (tailored TMS for autism in children) Onsite daycare Group/peer support NDIS services for children with intellectual and/or physical disabilities Allied health—dietetics and nutrition, post-partum recovery Community pharmacy Dentistry Onsite diagnostics
Chronic Disease	Early detection, prevention and holistic treatment—GPs, nurse practitioners and diabetes educators Specialists—oncology, cardiology, gastroenterology, orthopaedics, neurology, chiropractic Allied health—exercise physiology, physiotherapy, podiatry, dietetics and nutrition Psychology and counselling—emotional support Community services—emotional and physical health Community pharmacy—polypharmacy support Renal dialysis and nephrologists Sleep and respiratory support Wound management—including light therapy Dentistry Onsite diagnostics Disability support, including employment services No or low-cost health and wellbeing activities provided by internal and external partnerships Access to evidence-informed treatment—clinical trials

**Table 2:** Novel models within Morayfield Health Precinct (MHP).

Model	Description
Emerging Community-Based Impact Research Cluster	Patient experiences, including priority groups of low socio-economic and indigenous people Disease-specific—autism, cardiac/diabetes, chronic pain, foetal alcohol syndrome, post-traumatic stress disorder Workforce development—domestic violence, integrative (team-based) clinical care, urgent care, clinical trials Patient education—reading skills for patient–child relationships Health services—GP-led urgent care model Novel mental health treatment—neuromodulation to treat neurological and sleep disorders MedTech—mobile apps (practitioner and patient user interfaces), respiratory disease diagnosis, telehealth Research and health services co-design with community and clinical leaders within MHP
Integrated Chiropractic Care	Evidence-based chiropractor embedded in MHP with shared patient record and referral system to deliver team-based care (i.e., care plans)
Shared Services (Practice in a Practice)	Shared delivery of services within community pharmacy, including embedding of nurse practitioners, practice nurses, GPs and medical reception Shared reception, workforce and physical space where appropriate to enhance utilisation of health resources
Pandemic Preparedness	Quarantine capacity by design Collaborative governance structure to enable shared decisions to ensure continued undisrupted primary care access and safety Consistent communication internally and externally
Health Consumer Engagement	Participation within governance structures, including newly formed Human Research Ethics Committee Qualitative studies that involve health consumer participants and focus on their experience or perspective
Integration of Community-Based Services in Primary Care	Services that connect people with disabilities to meaningful work Relationship support, including elder abuse, domestic violence and parent–child relationships Antenatal and perinatal support No or low-cost health and wellbeing activities provided by external and internal partnerships
Workforce Development	Co-design of new multi-disciplinary urgent care qualifications Expansion of non-traditional practitioners into primary care delivery, such as paramedicine, exercise physiologists and occupational therapists Supporting occupational work experience in primary care for students studying nursing, midwifery, medicine, allied health and paramedicine

## Data Availability

Not applicable.

## References

[1] Malachowski C., Skopyk S., Toth K., MacEachen E. (2019). The integrated health hub (IHH) model: The evolution of a community based primary care and mental health centre. Community Ment. Health J..

[2] Trankle S.A., Usherwood T., Abbott P., Roberts M., Crampton M., Girgis C.M., Riskallah J., Chang Y., Saini J., Reath J. (2019). Integrating health care in Australia: A qualitative evaluation. BMC Health Serv. Res..

[3] World Health Organization (2015). WHO Global Strategy on People-Centred and Integrated Health Services: Interim Report.

[4] Busetto L. (2016). Great expectations: The implementation of integrated care and its contribution to improved outcomes for people with chronic conditions. Int. J. Integr. Care.

[5] OECD Health at a Glance 2019: OECD Indicators. https://www.oecd-ilibrary.org/social-issues-migration-health/health-at-a-glance-2019_4dd50c09-en.

[6] World Health Organization (2022). The Path towards Universal Health Coverage.

[7] Javanparast S., Roeger L., Reed R.L. (2021). Experiences of patients with chronic diseases of access to multidisciplinary care during COVID-19 in South Australia. Aust. Health Rev..

[8] Podubinski T., Townsin L., Thompson S.C., Tynan A., Argus G. (2021). Experience of healthcare access in Australia during the first year of the COVID-19 pandemic. Int. J. Environ. Res. Public Health.

[9] Williams E. (2017). $100 Million Dollar Morayfield Health Hub Will Open in October.

[10] HHDM (2022). 200,000 Registered Patients’ Celebration.

[11] Australian Bureau of Statistics (2021). Morayfield: 2021 Census All Persons QuickStats.

[12] Australian Bureau of Statistics (2021). Moreton Bay: 2021 Census All Persons QuickStats.

[13] Nationwide News (2021). HomeCo in Buying Spree Ahead of Health Property Launch.

[14] Metro North HHS (2017). Joint Health Assessment—Metro North Health.

[15] Queensland Statistician’s Office (2021). Queensland Regions Compared, Census 2021—qgso.qld.gov.au.

[16] News Corp Australia (2018). Morayfield’s Beating Heart.

[17] Thomas H., Best M., Mitchell G. (2020). Whole-person care in general practice: ‘The nature of whole-person care’. Aust. J. Gen. Pract..

[18] Heuzenroeder C. (2021). ‘You Just Can’t Get Help’: Patients Struggle with COVID-19 Barriers to Respiratory Care.

[19] Weaver C. (2021). Application of the WHO Integrated Person-Centred Care Framework (IPCC) in the development of a large Health Hub in the Northern Suburbs of Brisbane. Int. J. Integr. Care.

[20] Gullery C., Hamilton G. (2015). Towards integrated person-centred healthcare–the Canterbury journey. Future Hosp. J..

[21] McHugh M.D., Aiken L.H., Eckenhoff M.E., Burns L.R. (2016). Achieving kaiser permanente quality. Health Care Manag. Rev..

[22] Department of Health and Aged Care (2022). Spikevax (moderna) COVID-19 Vaccine Available in Pharmacies.

[23] HHDM (2023). COVID-19 Vaccination Clinic.

[24] TerryWhite Chemmart Morayfield (2022). Your Local, Trusted, Full Service Pharmacy.

[25] HHDM (2021). DR Posture—A Simple Problem Deserves a Simple Solution.

[26] Open Cities (2022). Start 2023 Right with Free & Low-Cost Health and Wellbeing Activities.

[27] Attwooll J. (2022). Budget in Detail: The Impact on General Practice.

[28] Cowling T.E., Ramzan F., Ladbrooke T., Millington H., Majeed A., Gnani S. (2016). Referral outcomes of attendances at general practitioner led urgent care centres in London, England: Retrospective analysis of hospital administrative data. Emerg. Med. J..

[29] Van den Heede K., Van de Voorde C. (2016). Interventions to reduce emergency department utilisation: A review of reviews. Health Policy.

[30] Winkler J.K., Sies K., Fink C., Toberer F., Enk A., Deinlein T., Haenssle H.A. (2020). Melanoma recognition by a deep learning convolutional neural network—performance in different melanoma subtypes and localisations. Eur. J. Cancer.

[31] Ladhams A., Patel S., Çetin M. (2022). Implementation of a novel digital diagnostic tool to support the assessment of respiratory disease in a COVID-19 fever clinic. BMJ Innov..

[32] Ezedinma U., Swierkowski P., Fjaagesund S. (2022). Outcomes from Individual Alpha Frequency Guided Repetitive Transcranial Magnetic Stimulation in Children with Autism Spectrum Disorder–A Retrospective Chart Review. Child Psychiatry Hum. Dev..

[33] Al Nemari M., Waterson J. (2022). The Introduction of Robotics to an Outpatient Dispensing and Medication Management Process in Saudi Arabia: Retrospective Review of a Pharmacy-led Multidisciplinary Six Sigma Performance Improvement Project. JMIR Hum. Factors.

[34] Momattin H., Arafa S., Momattin S., Rahal R., Waterson J. (2021). Robotic Pharmacy Implementation and Outcomes in Saudi Arabia: A 21-Month Usability Study. JMIR Hum. Factors.

[35] atWork Australia (2022). We Help People Find Meaningful Work.

[36] Peach Tree Perinatal Wellness (2022). Because Parenthood Isn’t Always Peachy.

[37] Brewer M.L., Flavell H.L., Trede F., Smith M. (2016). A scoping review to understand “leadership” in interprofessional education and practice. J. Interprof. Care.

[38] Forsyth C., Mason B. (2017). Shared leadership and group identification in healthcare: The leadership beliefs of clinicians working in interprofessional teams. J. Interprof. Care.

[39] Greenhalgh T., Jackson C., Shaw S., Janamian T. (2016). Achieving research impact through co-creation in community-based health services: Literature review and case study. Milbank Q..

[40] HHDM (2022). Health Hub Morayfield’s Inaugural 2022 REEC Seed Research Grant Recipients.

